# Preexisting hemodialysis and survival outcome in out-of-hospital cardiac arrest patients: Ulsan, South Korea

**DOI:** 10.3389/fmed.2025.1434543

**Published:** 2025-01-16

**Authors:** Song Yi Park, Sun Hyu Kim, Byungho Choi

**Affiliations:** ^1^Department of Emergency Medicine, College of Medicine, Dong-A University Hospital, Dong-A University, Busan, Republic of Korea; ^2^Department of Emergency Medicine, College of Medicine, Ulsan University Hospital, University of Ulsan, Ulsan, Republic of Korea

**Keywords:** cardiopulmonary resuscitation, out-of-hospital cardiac arrest, renal dialysis, acute kidney injury, treatment outcome

## Abstract

**Background:**

Although the incidence of sudden cardiac death is higher in hemodialysis (HD) patients, whether out-of-hospital cardiac arrest (OHCA) survival outcomes are poorer in this group remains unclear. This study aimed to assess the impact of HD on survival outcomes among adult nontraumatic OHCA patients and to compare these outcomes between HD and non-HD groups.

**Methods:**

This observational cohort study retrospectively analyzed data from adult nontraumatic OHCA patients in Ulsan, South Korea, from January 2017 through December 2022. Multivariable logistic regression analysis was applied to evaluate whether HD was a risk factor for survival in OHCA patients. Survival was compared between the two groups in unadjusted, balanced groups by propensity score matching (PSM) and inverse probability of the treatment weighting (IPWT).

**Results:**

The study included 2,489 patients (64 HD group and 2,425 non-HD group). Undergoing HD was not significantly associated with any return of spontaneous circulation (ROSC) (adjusted odds ratio [95% confidence interval], *p*-value, 1.648 [0.934–2.907], 0.085), survival to discharge (1.544 [0.734–3.250], 0.252), or neurological outcomes (0.394 [0.017–9.346], 0.564). There were also no significant differences observed in any ROSC (1.648 [0.934–2.907], 0.085), survival to discharge (1.544 [0.734–3.250], 0.252), or favorable neurological outcome (0.394 [0.017–9.346], 0.564) between the two unadjusted groups. The insignificant survival differences were persistently observed in the PSM group and IPWT group.

**Conclusion:**

Although HD may pose a risk factor for cardiac arrest, our study did not find a significant association with survival outcomes in OHCA patients. Additionally, no notable survival difference was observed between HD and non-HD groups. Therefore, resuscitation efforts in HD patients should not be underestimated.

## Introduction

Sudden cardiac death (SCD) is a significant cause of mortality among end-stage kidney disease (ESKD) patients (1). A systematic review reported that the annual incidence of SCD within the ESKD population undergoing hemodialysis (HD) ranges widely from 0.4 to 10.4% (2). However, it is difficult to estimate its incidence precisely due to difficulties involved in defining or classifying the term (2, 3). This challenge appears to be similarly reflected in the study that has documented SCD incidence within the HD population of South Korea (4).

The SCD among ESKD patients is thought to result from a complex interplay of multiple factors, including vascular calcifications, left ventricular hypertrophy, and arrhythmic triggers such as hyperkalemia, fluid overload, and abrupt changes in blood pressure during dialysis sessions (1). One proposed mechanism involves both myocardial susceptibility and an acute proarrhythmic event, which can lead to fatal arrhythmia such as ventricular tachycardia or fibrillation (5). Data from the US Renal Data System indicates that 40% of known deaths among dialysis patients are due to arrhythmias and cardiac arrest (3). Additionally, the presence of comorbidities such as diabetes mellitus, hypertension, and coronary artery disease further exacerbates the risk of SCD in HD patients (6, 7). Given the fatal arrhythmia require immediate rhythm intervention, the timing of emergency medical services (EMS) arrival is crucial for this patient population.

Out-of-hospital cardiac arrest (OHCA) is a critical situation distinguished by the abrupt loss of heart function, posing a substantial public health challenge worldwide. The frequency of OHCA cases managed by EMS varies widely across countries, with rates spanning from 30 to 97 per 100,000 individuals annually (8). In comparison, the survival outcomes for OHCA patients resuscitated by EMS remain globally at around 8% (8, 9). In Korea, the incidence of OHCAs assessed by EMS was 46.8 per 100,000 individuals in 2010, with a survival rate of OHCAs treated by EMS at 3.6% (10). However, a more recent study in 2015 reported a higher survival rate of 9.6%, with 1.9% of cases achieving favorable neurological outcomes (11).

According to the Korean Renal Data System, by 2019, a total of 108,873 patients were receiving renal replacement therapy for ESKD in Korea. Among these, 81,760 patients (75.1%) were undergoing HD, 5,960 patients (5.5%) were receiving peritoneal dialysis, and 21,153 patients (19.4%) had undergone kidney transplantation KT. The prevalence of HD in Korea in 2019 was approximately 1.58 per 1,000 population and has doubled since 2010, largely due to rapid population aging. Furthermore, Korea ranks sixth in the world for ESKD incidence, with an incidence rate of 1,816 new cases per million population. Currently, diabetes mellitus is the most common cause of ESKD in Korea, accounting for 48.4%, followed by hypertension as the second. The percentage of ESKD patients with kidney disease of unknown origin has remained above 10% in recent years, making it the third leading cause of ESKD (12).

While studies indicate that there is a higher incidence of SCD among patients undergoing HD, it remains unknown whether survival outcomes following OHCA are inferior in patients who were undergoing HD prior to the arrest compared to those who were not undergoing HD. This question is essential because various factors may influence OHCA survival outcomes, including patient-related factors (age, sex, concurrent medical conditions), as well as whether the event was witnessed and the location of the arrest. Additionally, bystander interventions (cardiopulmonary resuscitation (CPR) or an automated external defibrillator (AED) use) and EMS interventions (EMS processing duration or prehospital epinephrine administration, etc.) also play a significant role (13–15). A study in Taiwan found that, although undergoing HD patients exhibit an increased risk of OHCA, they demonstrated a higher chance of return of spontaneous circulation (ROSC) and enhanced short-term hospital outcomes than non-HD individuals (16).

There is currently a knowledge gap regarding whether undergoing HD status represents a significant risk factor for survival in OHCA patients. Addressing this gap is crucial to helping inform clinical decision-making and optimize patient care. Therefore, the research question of this study is whether undergoing HD is a factor related to survival outcomes in OHCA patients. The objective of this study is to investigate the association of undergoing HD with survival outcomes and compare survival outcomes among HD and non-HD patients.

## Methods

### Design and setting

This observational cohort study retrospectively analyzed data of adult nontraumatic OHCA patients in Ulsan, South Korea, from January 1st, 2017, through December 31st, 2022. The study aimed to determine whether undergoing HD poses a risk for survival among OHCA patients and to compare survival outcomes between OHCA patients undergoing HD (HD group) and those not undergoing HD (non-HD group). This study received approval from the Ethics Committee of Ulsan University Hospital (Reference Number: UUH-IRB-03-002) with an informed consent waiver. This study was conducted in accordance with the principles outlined in the Declaration of Helsinki.

Ulsan is located on the eastern coast of South Korea, with an area spanning 1,057.136 km^2^ and a population exceeding 1.1 million. As of 2022, Ulsan has 30 fire stations and a central dispatch center. The EMS system in Ulsan follows South Korea’s national EMS framework (17). The city’s EMS teams consist of two or three highly trained personnel, including at least one member who is an emergency medical technician (EMT). These individuals are certified registered nurses or possess the qualifications of levels 1 and 2 EMT, which are comparable to basic to intermediate levels EMTs in the USA. When suspected cardiac arrest occurs, it is standard protocol to dispatch multiple EMS teams (two or more) from fire stations to the scene for immediate on-site resuscitation. Patients are subsequently moved to the emergency department (ED) with continuous CPR during transit. EMS personnel are not authorized to stop CPR unless specific criteria are met, such as any ROSC, confirmation of definite death sign (livor mortis or rigor mortis), or presence of a do-not-resuscitate (DNR) order. The official death could be declared under the presence of physicians within the hospital EDs; physicians are generally not present in ambulances. Advanced resuscitation interventions are administered under the direct oversight of medical directors, who primarily consist of emergency physicians in the dispatch center (18, 19). After the patient arrives in the ED, resuscitation is performed according to the Advanced Cardiovascular Life Support guidelines by the American Heart Association.

### Patients

All patients that EMS personnel assessed as OHCA within Ulsan during the study period were included as the study population. Among them, (1) resuscitation being withheld or withdrawn based on the presence of death signs or a DNR order; (2) suspected arrest by intoxication, drowning, or trauma; and (3) individuals aged less than 18 years old were excluded. Study populations were stratified into the HD group if they underwent HD before the event of cardiac arrest. It was confirmed directly to have a functioning arteriovenous fistula (AVF) by EMS personnel and extracted from the dispatch record and prehospital patient care reports. The non-HD group was comprised of patients who were not in the HD group.

### Data collection

Data extraction was performed in two phases. Prehospital data were obtained from the Ulsan Fire Agency, which compiles reports on prehospital cardiac arrest cases following the Utstein OHCA template (20). These reports contain detailed information on survival-related factors, including patient history, comorbidities, and timelines of prehospital interventions. The data is standardized across all Korean Fire Agencies for quality control. The dataset, provided in Excel format, was de-identified, with patient information represented by EMS serial numbers. A field indicating the presence of a functioning AVF, checked by EMS personnel to secure an intravenous line, was used to classify patients into two groups. For the hospital data, survival outcomes were obtained by contacting the 17 EDs in the region where patients were transported, using the time of visit and EMS serial number. This was facilitated by the provisions of the Information Disclosure Act, which allows hospitals to disclose patient treatment outcomes for research purposes. The likelihood of misreporting survival status by hospital staff is considered very low. The study population was limited to those cases occurring within Ulsan; however, a few patients whose OHCA occurred near the Ulsan border were transferred to EDs in neighboring areas. In such cases, we traced their outcomes.

Variables were collected according to patient, bystander, EMS, and hospital-related factors. Patient-related variables comprised age, sex, concurrent medical conditions (diabetes mellitus, hypertension, cerebrovascular disease, cardiovascular disease, pulmonary disease, liver disease, renal failure, and malignancy), whether the event was witnessed (witnessed arrest), and where the event occurred (arrest location). Bystander-related variables comprised bystander CPR administration (bystander CPR) and bystander AED use (bystander AED). EMS-related variables included initial rhythm analysis at the scene (initial rhythm), types of advanced airway devices (advanced airway), mechanical compression device use (mechanical compression), epinephrine use, and EMS process duration. The EMS process duration was disaggregated into three intervals: the duration from the EMS team’s dispatch from the fire station to their arrival at the scene (response time interval, RTI), the duration of performing resuscitation at the scene (on-scene time interval, STI), and the duration from departing the scene to transporting the patient to the ED (transport time interval, TTI). Hospital-related variables comprised in-hospital interventions. In-hospital intervention included the implementation of targeted temperature management (TTM), coronary angiography (CAG), and extracorporeal membrane oxygenation (ECMO).

### Outcome

All patients were traced until discharge, including transfers to other hospitals. Survival outcomes were classified and collected as any ROSC, survival to discharge, and neurological outcomes. Any ROSC was defined as the ROSC at any point during the resuscitation attempt after the patient’s arrival at the ED, regardless of the patient’s final survival outcome. Survival to discharge was defined as being alive at the time point of hospital discharge. Neurological outcomes status was assessed through the Cerebral Performance Categories scale scores reported in discharge records, with score of 1 and 2 indicating favorable neurological outcomes (21). The primary outcome was set as an association between undergoing HD and OHCA survival outcomes. The secondary outcome was set as a comparison of the survival outcomes between the HD and non-HD populations.

### Statistical analysis

Categorical variables were presented using frequencies with percentages. Continuous variables were delineated using the mean with standard deviation (SD) or the median with interquartile range (IQR) according to the normality tests using the Kolmogorov–Smirnov and Shapiro–Wilk tests, respectively. In cases in which the normality of the two groups differed, both mean with SD and median with IQR were reported.

The comparison of the two groups was performed after balancing the discrepancy in sample size between the two groups with propensity score matching (PSM) and inverse probability of treatment weighting (IPTW) methods (22). PSM included all variables except survival outcome. Subsequently, comparisons were conducted in the unadjusted group, PSM group, and IPTW group. For continuous variables, either an Independent t-test or a Mann–Whitney test was performed according to the normality assessment. For categorical variables, a Chi-square test or Fisher’s exact test was applied as appropriate to each specific variable.

In assessing the association between survival outcomes and undergoing HD and comparing the survival outcomes between the HD and non-HD groups, multivariable logistic regression analysis was conducted, providing odds ratios (OR) along with a 95% confidence interval (CI). Predictor variables encompassed patient-related factors (age, sex, concurrent medical conditions, witness arrest, arrest location), bystander-related factors (bystander CPR, bystander AED), EMS-related factors (initial rhythm, EMS process duration, advanced airway, mechanical compression, epinephrine use), and hospital-related factors (implementation of TTM, CAG, ECMO). Backward selection was employed to construct the final adjusted model, and the goodness of fit was assessed using the Hosmer-Lemeshow test. Statistical significance was set at *p* < 0.05. All statistical tests were drawn using SAS (v. 9.4; SAS Institute Inc., Cary, NC, USA).

## Results

**Figure 1:**
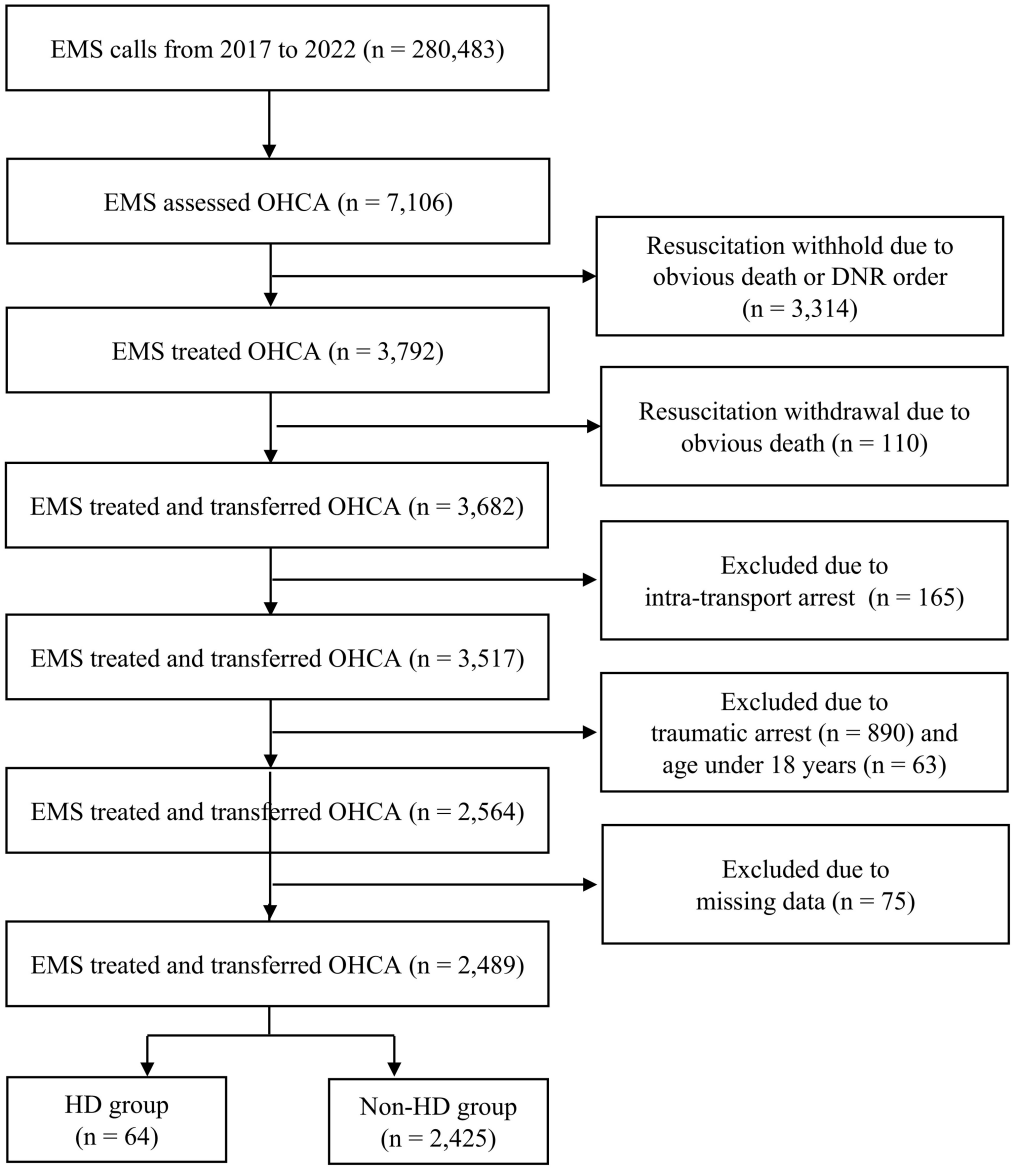
Study flow diagram. EMS, emergency medical services; OHCA, out-of-hospital cardiac arrest; DNR, do-not-resuscitate; HD, hemodialysis.

Throughout the study period, Ulsan recorded a total of 280,483 EMS calls. Among these, EMS personnel assessed 7,106 patients as OHCA. They initiated resuscitation for 3,792 patients while withholding it for the remaining 3,314 due to evident signs of death or existing DNR orders. During resuscitation, 110 patients were excluded upon confirmation of death (resuscitation withdrawal). Ultimately, 3,682 patients were transported to EDs. Among them, 165 patients were excluded due to intra-transport arrest, 890 were excluded due to presumed traumatic arrest, and 63 were excluded due to being under 18 years old. An additional 75 patients were excluded due to missing data. Consequently, the current study included a total of 2,489 patients. The HD group comprised 64 patients, whereas the non-HD group consisted of 2,425 patients (Figure 1).

### Patient characteristics

Table 1 displays the characteristics of the HD and non-HD groups. There were no notable disparities in age or sex distribution between the two groups. However, significant differences were observed in concurrent medical conditions, with the HD group showing a higher proportion of diabetes mellitus (54.7% vs. 18.5%, *p* < 0.001) and a lower proportion of malignancy (3.1% vs. 11.7%, 0.034) compared to the non-HD group. No significant differences were seen in the bystander, EMS, or hospital-related variables (Table 1). The comparisons between the HD and non-HD groups after 1:5 PSM and IPTW, were presented in Tables 2, 3. They demonstrate balanced demographic and clinical characteristics between the groups (Tables 2, 3).

**Table 1:** Characteristics of adult nontraumatic OHCA patients.

	HD group	Non-HD group	*p*-value
	(*n* = 64)	(*n* = 2,425)	
*Patient-related variables*			
Age, years, mean (SD)	67.5 (11.2)	69.0 (15.7)	0.284
Age, years, median (Q1–Q3)	66.5 (61.0–75.8)	72.0 (58.0–81.0)	
Sex (male)	37 (57.8)	1,512 (62.4)	0.460
Concurrent medical conditions			
Hypertension	22 (34.4)	614 (25.3)	0.101*
Diabetes mellitus	35 (54.7)	448 (18.5)	<0.001*
Cerebrovascular disease	2 (3.1)	157 (6.5)	0.434
Cardiovascular disease	11 (17.2)	367 (15.1)	0.651
Pulmonary disease	1 (1.6)	153 (6.3)	0.182
Liver disease	2 (3.1)	45 (1.9)	0.342
Malignancy	2 (3.1)	283 (11.7)	0.034*
Witnessed arrest, witnessed	30 (46.9)	1,057 (43.6)	0.601
Arrest location, public	4 (6.3)	457 (18.8)	0.010*
*Bystander-related variables*			
Bystander CPR, performed	43 (67.2)	1,493 (61.6)	0.361
Bystander AED, applied	1 (1.6)	69 (2.8)	1.000
*EMS-related variables*			
Initial rhythm, shockable	9 (14.1)	408 (16.8)	0.559
EMS process duration, minutes			
RTI mean (SD)	7.6 (3.4)	7.8 (3.9)	0.746
RTI median (Q1–Q3)	7.0 (5.0–9.0)	7.0 (5.0–9.0)	
STI mean (SD)	14.5 (4.9)	14.4 (5.4)	0.933
STI median (Q1–Q3)	14.0 (12.0–18.0)	14.0 (11.0–17.0)	
TTI mean (SD)	6.1 (5.0)	6.3 (5.2)	0.746
TTI median (Q1–Q3)	5.0 (3.0–6.8)	5.0 (3.0–8.0)	
Advanced airway			
No advanced airway	5 (7.8)	316 (13.0)	0.107
Tracheal intubation	12 (18.8)	272 (11.2)	
I-gel/supraglottic airway	47 (73.4)	1837 (75.8)	
Mechanical compression, applied	39 (60.9)	1,213 (50.0)	0.085
Epinephrine use	10 (15.6)	359 (14.8)	0.855
*Hospital-related variables*			
TTM	0 (0.0)	30 (1.2)	1.000
CAG	4 (6.3)	226 (9.3)	0.403
ECMO	0 (0.0)	38 (1.6)	0.624
*Survival outcomes*			
Any ROSC	25 (39.1)	777 (32.0)	0.235
Survival to discharge	11 (17.2)	363 (15.0)	0.624
Neurological outcome, favorable	2 (3.1)	143 (5.9)	0.584

The variables are presented as numbers (percentages). HD, Hemodialysis; SD, Standard deviation; CPR, Cardiopulmonary resuscitation; AED, Automated external defibrillator; RTI, Response time interval; STI, Scene time interval; TTI, Transport time interval; TTM, Targeted temperature management; CAG, Coronary angiography, ECMO, Extracorporeal membrane oxygenation; ROSC, Return of spontaneous circulation. ^*^ Indicates that the *p*-value is less than 0.05.

**Table 2:** Characteristics of adult nontraumatic OHCA patients after 1:5 propensity score matching.

	HD group	Non-HD group	*p*-value
	(*n* = 58)	(*n* = 290)	
*Patient-related variables*			
Age, years, mean (SD)	68.1 (11.3)	69.9 (15.2)	0.284
Age, years, median (Q1–Q3)	66.5 (61.0–76.0)	72.0 (59.0–82.0)	
Sex (male)	33 (56.9)	152 (52.4)	0.460
Concurrent medical conditions			
Hypertension	19 (32.8)	114 (39.3)	0.349
Diabetes mellitus	29 (50.0)	152 (52.4)	0.737
Cerebrovascular disease	2 (3.4)	9 (3.1)	1.000
Cardiovascular disease	10 (17.2)	48 (16.6)	0.898
Pulmonary disease	1 (1.7)	5 (1.7)	1.000
Liver disease	2 (3.4)	2 (0.7)	0.131
Malignancy	2 (3.4)	6 (2.1)	0.625
Witnessed arrest, witnessed	26 (44.8)	123 (42.4)	0.735
Arrest location, public	4 (6.9)	20 (6.9)	1.000
*Bystander-related variables*			
Bystander CPR, performed	38 (65.5)	186 (64.1)	0.841
Bystander AED, applied	1 (1.7)	6 (2.1)	1.000
*EMS-related variables*			
Initial rhythm, shockable	8 (13.8)	42 (14.5)	0.891
EMS process duration, minutes			
RTI mean (SD)	7.8 (3.4)	7.7 (3.8)	0.870
RTI median (Q1–Q3)	7.0 (5.0–9.0)	7.0 (5.0–9.0)	
STI mean (SD)	14.5 (5.1)	14.5 (5.3)	0.944
STI median (Q1–Q3)	14.0 (12.0–18.0)	14.0 (11.0–17.0)	
TTI mean (SD)	6.1 (5.2)	6.5 (5.6)	0.608
TTI median (Q1–Q3)	4.5 (3.0–6.0)	5.0 (3.0–8.0)	
Advanced airway			
No advanced airway	5 (8.6)	26 (9.0)	0.954
Tracheal intubation	7 (12.1)	39 (13.4)	
I-gel/supraglottic airway	46 (79.3)	225 (77.6)	
Mechanical compression, applied	34 (58.6)	158 (54.5)	0.563
Epinephrine use	8 (13.8)	51 (17.6)	0.482
*Hospital-related variables*			
TTM	0 (0.0)	0 (0.0)	
CAG	4 (6.9)	20 (6.9)	1.000
ECMO	0 (0.0)	0 (0.0)	
*Survival outcomes*			
Any ROSC	22 (37.9)	84 (29.0)	0.176
Survival to discharge	9 (15.5)	36 (12.4)	0.520
Neurological outcome, favorable	2 (3.4)	12 (4.1)	1.000

The variables are presented as numbers (percentages). HD, Hemodialysis; SD, Standard deviation; CPR, Cardiopulmonary resuscitation; AED, Automated external defibrillator; RTI, Response time interval; STI, Scene time interval; TTI, Transport time interval; TTM, Targeted temperature management; CAG, Coronary angiography, ECMO, Extracorporeal membrane oxygenation; ROSC, Return of spontaneous circulation. PSM included all variables except survival outcome.

**Table 3:** Characteristics of adult nontraumatic OHCA patients after inverse probability of treatment weighting.

	HD group	Non-HD group	*p*-value
	(*n* = 64)	(*n* = 2,425)	
*Patient-related variables*			
Age, years, mean (SD)	69.3 (10.5)	69.0 (15.7)	0.840
Age, years, median (Q1 – Q3)	69.0 (62.0–76.0)	72.0 (58.0–81.0)	
Sex (male)	37 (57.4)	1,509 (62.2)	0.429
Concurrent medical conditions			
Hypertension	14 (21.8)	619 (25.5)	0.499
Diabetes mellitus	18 (28.3)	470 (19.4)	0.075
Cerebrovascular disease	3 (5.2)	155 (6.4)	0.710
Cardiovascular disease	10 (14.9)	368 (15.2)	0.955
Pulmonary disease	2 (2.6)	150 (6.2)	0.239
Liver disease	2 (2.4)	46 (1.9)	0.781
Malignancy	3 (5.2)	278 (11.5)	0.121
Witnessed arrest, witnessed	30 (47.5)	1,059 (43.7)	0.544
Arrest location, public	7 (10.5)	449 (18.5)	0.101
*Bystander-related variables*			
Bystander CPR, performed	38 (60.1)	1,496 (61.7)	0.790
Bystander AED, applied	0 (0.4)	68 (2.8)	0.254
*EMS-related variables*			
Initial rhythm, shockable	8 (12.8)	406 (16.7)	0.401
EMS process duration, minutes			
RTI mean (SD)	7.7 (3.0)	7.7 (3.9)	0.806
RTI median (Q1–Q3)	7.0 (5.0–9.0)	7.0 (5.0–9.0)	
STI mean (SD)	13.5 (4.9)	14.4 (5.4)	0.182
STI median (Q1–Q3)	13.0 (10.0–17.0)	14.0 (11.0–17.0)	
TTI mean (SD)	6.0 (4.9)	6.3 (5.2)	0.695
TTI median (Q1–Q3)	5.0 (3.0–7.0)	5.0 (3.0–8.0)	
Advanced airway			
No advanced airway	8 (13.1)	313 (12.9)	0.857
Tracheal intubation	6 (9.2)	276 (11.4)	
I-gel/supraglottic airway	50 (77.7)	1836 (75.7)	
Mechanical compression, applied	31 (48.6)	1,219 (50.3)	0.791
Epinephrine use	7 (10.2)	359 (14.8)	0.302
*Hospital-related variables*			
TTM	0 (0.0)	29 (1.2)	0.377
CAG	4 (6.9)	224 (9.2)	0.524
ECMO	0 (0.0)	37 (1.5)	0.319
*Survival outcomes*			
Any ROSC	24 (37.8)	775 (32.0)	0.327
Survival to discharge	8 (13.2)	362 (14.9)	0.708
Neurological outcome, favorable	2 (3.8)	142 (5.9)	0.484

The variables are presented as numbers (percentages). HD, Hemodialysis; SD, Standard deviation; CPR, Cardiopulmonary resuscitation; AED, Automated external defibrillator; RTI, Response time interval; STI, Scene time interval; TTI, Transport time interval; TTM, Targeted temperature management; CAG, Coronary angiography, ECMO, Extracorporeal membrane oxygenation; ROSC, Return of spontaneous circulation.

### Factors associated with survival outcomes

Table 4 presents the factors found to be associated with any ROSC in the study population. Liver disease (adjusted OR = 2.105, *p* = 0.023), witnessed arrest (1.836, <0.001), and initial shockable rhythm (1.782, <0.001) were all associated with higher odds of any ROSC. Conversely, older age (0.986, <0.001), longer STI (0.977, 0.027), and mechanical compression (0.676, <0.001) were associated with lower odds of any ROSC. However, undergoing HD (1.648, 0.085) itself did not show a significant association with any ROSC.

**Table 4:** Factors associated with any ROSC in adult nontraumatic OHCA patients: multivariable logistic regression analysis.

	Unadjusted		Adjusted	
	OR (95% CI)	*p*-value	OR (95% CI)	*p*-value
Non-HD	1	reference	1	reference
HD	1.360 (0.817 ~ 2.263)	0.237	1.648 (0.934 ~ 2.907)	0.085
*Patient-related variables*				
Age, years	1.360 (0.817 ~ 2.263)	0.237	0.986 (0.980 ~ 0.993)	<0.001*
Sex, female	1	reference	1.000	reference
Sex, male	1.257 (1.055 ~ 1.498)	0.011	0.815 (0.658 ~ 1.009)	0.061
Concurrent medical conditions *(ref = non)*				
Hypertension	0.944 (0.778 ~ 1.146)	0.560	1.065 (0.832 ~ 1.365)	0.617
Diabetes mellitus	0.839 (0.675 ~ 1.042)	0.113	0.928 (0.707 ~ 1.217)	0.589
Cerebrovascular disease	0.716 (0.497 ~ 1.033)	0.074	0.916 (0.607 ~ 1.382)	0.675
Cardiovascular disease	1.138 (0.904 ~ 1.434)	0.272	1.171 (0.886 ~ 1.549)	0.267
Pulmonary disease	0.724 (0.500 ~ 1.049)	0.088	1.163 (0.780 ~ 1.734)	0.459
Liver disease	1.196 (0.656 ~ 2.182)	0.559	2.105 (1.109 ~ 3.993)	0.023*
Malignancy	0.563 (0.420 ~ 0.754)	0.000	0.838 (0.604 ~ 1.164)	0.292
Witnessed arrest, witnessed	2.309 (1.945 ~ 2.740)	<0.0001	1.836 (1.497 ~ 2.252)	< 0.001*
Arrest location, public	2.345 (1.907 ~ 2.883)	<0.0001	1.233 (0.936 ~ 1.626)	0.136
*Bystander-related variables*				
Bystander CPR, performed	1.237 (1.039 ~ 1.474)	0.017	0.959 (0.779 ~ 1.180)	0.691
Bystander AED, applied	1.100 (0.667 ~ 1.816)	0.708	1.078 (0.614 ~ 1.895)	0.793
*EMS-related variables*				
Initial rhythm, nonshockable	1	reference	1.000	reference
Initial rhythm, shockable	4.920 (3.940 ~ 6.143)	<0.0001	1.782 (1.322 ~ 2.402)	0.000*
EMS process duration, minutes				
RTI	1.257 (1.055 ~ 1.498)	0.011	0.977 (0.950 ~ 1.004)	0.089
STI	0.944 (0.778 ~ 1.146)	0.560	0.977 (0.957 ~ 0.997)	0.027*
TTI	0.839 (0.675 ~ 1.042)	0.113	1.013 (0.991 ~ 1.035)	0.239
Advanced airway				
No advanced airway	reference	0.155	reference	0.778
Tracheal intubation	0.890 (0.637 ~ 1.244)	0.497	1.093 (0.721 ~ 1.658)	0.675
I-gel/supraglottic airway	0.793 (0.619 ~ 1.016)	0.066	1.121 (0.816 ~ 1.541)	0.480
Mechanical compression, applied	0.487 (0.410 ~ 0.578)	<0.0001	0.676 (0.545 ~ 0.839)	0.000*
Epinephrine use	1.061 (0.839 ~ 1.342)	0.621	1.217 (0.911 ~ 1.627)	0.184
*Hospital-related variables*				
TTM	>999.999 (<0.001~ > 999.999)	0.953	>999.999 (<0.001~ > 999.999)	0.985
CAG	>999.999 (<0.001~ > 999.999)	0.945	>999.999 (<0.001~ > 999.999)	0.963
ECMO	>999.999 (<0.001~ > 999.999)	0.966	>999.999 (<0.001~ > 999.999)	0.985

The variables are presented as numbers (percentages). HD, Hemodialysis; SD, Standard deviation; CPR, Cardiopulmonary resuscitation; AED, Automated external defibrillator; RTI, Response time interval; STI, Scene time interval; TTI, Transport time interval; TTM, Targeted temperature management; CAG, Coronary angiography, ECMO, Extracorporeal membrane oxygenation; ROSC, Return of spontaneous circulation. ^*^ Indicates that the p-value is less than 0.05.

Table 5 identifies the factors associated with survival to discharge. Cardiovascular disease (1.595, 0.010), witnessed arrest (1.779, <0.001), arrest in public (1.466, 0.018), initial shockable rhythm (2.379, <0.001), and CAG (6.577, <0.001) were all associated with favorable outcomes. Conversely, older age (0.972, <0.001), malignancy (0.569, 0.044), mechanical compression (0.525, <0.001), and ECMO (0.145, <0.001) were associated with unfavorable survival to discharge. However, undergoing HD (1.544, 0.252) itself did not show a significant association with survival to discharge.

**Table 5:** Factors associated with survival to hospital discharge in adult nontraumatic OHCA patients: multivariable logistic regression analysis.

	Unadjusted		Adjusted	
	OR (95% CI)	*p*-value	OR (95% CI)	*p*-value
Non-HD	1	reference	1	reference
HD	1.179 (0.610 ~ 2.279)	0.624	1.544 (0.734 ~ 3.250)	0.252
*Patient-related variables*				
Age, years	0.716 (0.497 ~ 1.033)	0.074	0.972 (0.963 ~ 0.980)	<0.001*
Sex, female	1	reference	1.000	reference
Sex, male	1.750 (1.371 ~ 2.233)	< 0.001	0.965 (0.718 ~ 1.298)	0.816
Concurrent medical condition *(ref = non)*				
Hypertension	0.806 (0.620 ~ 1.047)	0.107	1.042 (0.747 ~ 1.453)	0.809
Diabetes mellitus	0.747 (0.555 ~ 1.006)	0.055	1.008 (0.697 ~ 1.459)	0.965
Cerebrovascular disease	0.572 (0.332 ~ 0.985)	0.044	0.867 (0.472 ~ 1.591)	0.645
Cardiovascular disease	1.322 (0.992 ~ 1.763)	0.057	1.595 (1.120 ~ 2.273)	0.010*
Pulmonary disease	0.461 (0.253 ~ 0.839)	0.011	0.985 (0.523 ~ 1.856)	0.962
Liver disease	0.669 (0.263 ~ 1.703)	0.400	1.129 (0.403 ~ 3.158)	0.817
Malignancy	0.328 (0.199 ~ 0.543)	<0.001	0.569 (0.329 ~ 0.986)	0.044*
Witnessed arrest, witnessed	2.473 (1.971 ~ 3.102)	<0.001	1.779 (1.353 ~ 2.340)	<0.001*
Arrest location, public	3.344 (2.628 ~ 4.254)	<0.001	1.466 (1.069 ~ 2.011)	0.018*
*Bystander-related variables*				
Bystander CPR, performed	1.478 (1.167 ~ 1.872)	0.001	0.991 (0.749 ~ 1.311)	0.951
Bystander AED, applied	1.176 (0.625 ~ 2.211)	0.616	0.885 (0.425 ~ 1.843)	0.744
*EMS-related variables*				
Initial rhythm, nonshockable	1	reference	1.000	reference
Initial rhythm, shockable	7.353 (5.762 ~ 9.382)	<0.001	2.379 (1.718 ~ 3.295)	<0.001*
EMS process duration, minutes				
RTI	1.138 (0.904 ~ 1.434)	0.272	0.988 (0.953 ~ 1.024)	0.495
STI	0.724 (0.500 ~ 1.049)	0.088	0.981 (0.955 ~ 1.007)	0.153
TTI	1.196 (0.656 ~ 2.182)	0.559	1.020 (0.996 ~ 1.044)	0.107
Advanced airway				
No advanced airway	reference	<0.001	reference	0.542
Tracheal intubation	0.569 (0.375 ~ 0.864)	0.008	0.788 (0.471 ~ 1.316)	0.362
I-gel/supraglottic airway	0.518 (0.388 ~ 0.692)	<0.001	0.819 (0.564 ~ 1.190)	0.294
Mechanical compression, applied	0.315 (0.247 ~ 0.401)	<0.001	0.525 (0.391 ~ 0.705)	<0.001*
Epinephrine use	0.650 (0.459 ~ 0.921)	0.015	0.749 (0.494 ~ 1.136)	0.174
*Hospital-related variables*				
TTM	5.855 (2.838 ~ 12.082)	<0.001	1.470 (0.571 ~ 3.785)	0.425
CAG	14.098 (10.459 ~ 19.004)	<0.001	6.577 (4.456 ~ 9.705)	<0.001*
ECMO	1.774 (0.833 ~ 3.778)	0.137	0.145 (0.061 ~ 0.346)	<0.001*

The variables are presented as numbers (percentages). HD, Hemodialysis; SD, Standard deviation; CPR, Cardiopulmonary resuscitation; AED, Automated external defibrillator; RTI, Response time interval; STI, Scene time interval; TTI, Transport time interval; TTM, Targeted temperature management; CAG, Coronary angiography, ECMO, Extracorporeal membrane oxygenation; ROSC, Return of spontaneous circulation. ^*^ Indicates that the *p*-value is less than 0.05.

Table 6 presents the factors associated with neurological outcomes. Cardiovascular disease (3.783, <0.001), witnessed arrest (2.204, 0.018), initial shockable rhythm (9.006, <0.001), longer TTI (1.049, 0.019), and CAG (53.894, <0.001) were associated with increased likelihood of neurological outcomes. Conversely, older age (0.954, <0.001), all types of the advanced airway (tracheal intubation, 0.309, 0.033, I-gel/supraglottic airway, 0.484, 0.051), mechanical compression (0.144, <0.001), TTM (0.238, 0.029), and ECMO (0.139, <0.001) were associated with a decreased likelihood of favorable neurological outcomes. Undergoing HD (0.394, 0.564) did not demonstrate a significant association with neurological outcomes.

**Table 6:** Factors associated with favorable neurological outcome in adult nontraumatic OHCA patients: multivariable logistic regression analysis.

	Unadjusted		Adjusted	
	OR (95% CI)	*p*-value	OR (95% CI)	*p*-value
Non-HD	1	reference	1	reference
HD	0.515 (0.125 ~ 2.126)	0.359	0.394 (0.017 ~ 9.346)	0.564
*Patient-related variables*				
Age, years	0.563 (0.420 ~ 0.754)	0.000	0.954 (0.934 ~ 0.975)	<0.001*
Sex, female	1.000	reference	1.000	reference
Sex, male	3.073 (1.981 ~ 4.765)	<0.001	0.878 (0.411 ~ 1.874)	0.736
Concurrent medical condition *(ref = non)*				
Hypertension	0.748 (0.495 ~ 1.130)	0.168	1.232 (0.586 ~ 2.590)	0.582
Diabetes mellitus	0.573 (0.346 ~ 0.948)	0.030	1.226 (0.496 ~ 3.031)	0.659
Cerebrovascular disease	0.401 (0.146 ~ 1.097)	0.075	0.968 (0.200 ~ 4.676)	0.968
Cardiovascular disease	1.707 (1.139 ~ 2.559)	0.010	3.783 (1.810 ~ 7.905)	0.000*
Pulmonary disease	<0.001 (<0.001~ > 999.999)	0.970	<0.001 (<0.001~ > 999.999)	0.972
Liver disease	0.347 (0.048 ~ 2.534)	0.297	1.013 (0.015 ~ 68.888)	0.995
Malignancy	0.263 (0.107 ~ 0.648)	0.004	1.439 (0.367 ~ 5.649)	0.602
Witnessed arrest, witnessed	4.765 (3.205 ~ 7.087)	< 0.001	2.204 (1.146 ~ 4.240)	0.018*
Arrest location, public	4.807 (3.410 ~ 6.776)	< 0.001	1.273 (0.674 ~ 2.404)	0.457
*Bystander-related variables*				
Bystander CPR, performed	2.603 (1.718 ~ 3.942)	<0.001	1.089 (0.562 ~ 2.113)	0.800
Bystander AED, applied	0.979 (0.352 ~ 2.725)	0.968	1.106 (0.235 ~ 5.206)	0.899
*EMS-related variables*				
Initial rhythm, nonshockable	1.000	reference	1.000	reference
Initial rhythm, shockable	43.917 (26.968 ~ 71.520)	<0.001	9.006 (4.561 ~ 17.786)	<0.001*
EMS process duration, minutes				
RTI	2.309 (1.945 ~ 2.740)	< 0.001	0.943 (0.851 ~ 1.045)	0.264
STI	2.345 (1.907 ~ 2.883)	< 0.001	1.016 (0.962 ~ 1.073)	0.561
TTI	1.237 (1.039 ~ 1.474)	0.017	1.049 (1.008 ~ 1.093)	0.019
Advanced airway				
No advanced airway	reference	<0.001	reference	0.061
Tracheal intubation	0.293 (0.151 ~ 0.569)	0.000	0.309 (0.105 ~ 0.910)	0.033
I-gel/supraglottic airway	0.337 (0.229 ~ 0.496)	<0.001	0.484 (0.233 ~ 1.003)	0.051
Mechanical compression, applied	0.088 (0.049 ~ 0.156)	<0.001	0.144 (0.064 ~ 0.325)	<0.001*
Epinephrine use	0.455 (0.244 ~ 0.851)	0.014	0.586 (0.219 ~ 1.564)	0.286
*Hospital-related variables*				
TTM	4.173 (1.678 ~ 10.375)	0.002	0.238 (0.065 ~ 0.866)	0.029*
CAG	109.820 (67.576 ~ 178.471)	<0.001	53.894 (28.279 ~ 102.713)	<0.001*
ECMO	4.504 (2.026 ~ 10.011)	0.000	0.139 (0.049 ~ 0.397)	0.000*

The variables are presented as numbers (percentages). HD, Hemodialysis; SD, Standard deviation; CPR, Cardiopulmonary resuscitation; AED, Automated external defibrillator; RTI, Response time interval; STI, Scene time interval; TTI, Transport time interval; TTM, Targeted temperature management; CAG, Coronary angiography, ECMO, Extracorporeal membrane oxygenation; ROSC, Return of spontaneous circulation. ^*^ Indicates that the *p*-value is less than 0.05.

### Comparison of survival outcomes between HD and non-HD group

No differences were observed in any ROSC (1.648, 0.085), survival to discharge (1.544, 0.252), or neurological outcome (0.394, 0.564) between the two groups in the unadjusted study population. The insignificant survival differences were persistently observed in the PSM group and IPWT group (Table 7).

**Table 7:** Comparison of survival outcomes in adult nontraumatic OHCA patients in HD and Non-HD groups.

	Total	Any ROSC	Adjusted OR (95% CI)	*p*-value	Survival to hospital discharge	Adjusted OR (95% CI)	*p*-value	Neurological outcome	Adjusted OR (95% CI)	*p*-value
Study population										
Non-HD group	2,425	777 (32.0%)	Reference		363 (15.0%)	Reference		143 (5.9%)	Reference	
HD group	64	25 (39.1%)	1.648 (0.934 ~ 2.907)	0.085	11 (17.2%)	1.544 (0.734 ~ 3.250)	0.252	2 (3.1%)	0.394 (0.017 ~ 9.346)	0.564
Propensity score matching group										
Non-HD group	290	84 (29.0%)	Reference		36 (12.4%)	Reference		12 (4.1%)	Reference	
HD group	58	22 (37.9%)	1.499 (0.832 ~ 2.698)	0.177	9 (15.5%)	1.296 (0.587 ~ 2.861)	0.521	2 (3.4%)	0.827 (0.180 ~ 3.799)	0.808
Inverse propensity weighting group										
Non-HD group	2,425	775 (32.0%)	Reference		362 (14.9%)	Reference		142 (5.9%)	Reference	
HD group	64	24 (37.8%)	1.291 (0.773 ~ 2.156)	0.328	8 (13.2%)	0.870 (0.419 ~ 1.807)	0.708	2 (3.8%)	0.632 (0.173 ~ 2.310)	0.488

OHCA, out-of-hospital cardiac arrest; HD, hemodialysis; ROSC, return of spontaneous circulation; OR, odds ratio; CI, confidence interval.

## Discussion

This study aimed to evaluate how undergoing HD impacts survival outcomes among adult nontraumatic OHCA patients and whether there are any differences in survival outcomes between HD and non-HD groups. During a six-year observation period, 2.6% of adult nontraumatic OHCA patients had undergone HD before cardiac arrest. Despite this, undergoing HD was not independently associated with achieving any ROSC, survival to discharge, or favorable neurological outcomes. Furthermore, the survival outcome in the HD group did not significantly differ from that of the non-HD group. Instead, we identified several key factors that were significantly associated with survival outcomes, including age, cardiovascular disease, witnessed status of arrest, and initial shockable rhythm.

In our study, HD status was not a significant factor associated with survival outcomes. We identified repetitive factors consistently associated with the survival outcomes of the study population, including age, witnessed arrest, and initial shockable rhythms. These factors are consistent with the findings of previous OHCA research (23, 24). Moreover, these factors are well-known predictors of good survival outcomes. Considering particular significance as cardiac arrest pathophysiology in ESKD patients undergoing HD is frequently attributed to terminal arrhythmias, this fatal arrhythmia could be managed by prompt EMS activation and AED applications. Prompt EMS activation and AED applications could be possible and beneficial in the case of witnessed arrest and initial shockable rhythms in undergoing HD patients.

However, an interesting finding of our research is the association between cardiovascular disease and favorable survival outcomes. Cardiovascular disease emerged as a significant predictor of favorable survival outcomes. It has been widely acknowledged that cardiovascular disease poses a risk factor for OHCA (25, 26). Regarding this finding, we hypothesize that individuals with cardiovascular disease may have been more vigilant, which may have led them to seek more prompt help from the EMS system during emergencies. Moreover, the hospital’s emergency responses, such as CAG and percutaneous coronary intervention, may have significantly contributed to improving outcomes for these patient populations.

We attribute the lack of any observed association between undergoing HD and survival outcome in OHCA patients to post-arrest acute kidney injury (AKI) in many non-HD patients. Study have shown that post-arrest AKI affects over 50% of cardiac arrest patients (27). Another study revealed that 48.3% of post-arrest patients were observed to have AKI stage 3 (28). Further, AKI is associated with unfavorable neurological outcomes at six months among OHCA patients with TTM (29). There is a lack of similar studies, which makes it challenging to directly compare our findings with previous research. However, the findings of our study were consistent with previous research. A study from Taiwan reported that ESKD patients had a higher likelihood of ROSC and non-inferior hospital survival outcome rates compared to non-ESKD patients (30). While undergoing HD itself may be seen as a risk factor for cardiac arrest, our current study did not find it to be associated with survival outcomes of OHCA.

We ascribe the lack of difference in survival indicators between HD and non-HD groups to the multiple factors, beyond undergoing HD status, that are likely to influence survival in OHCA patients. One study conducted in Denmark found that the absence of concurrent medical conditions did not correlate independently with OHCA outcomes (31). Similarly, a study in the Netherlands found that certain resuscitation-related factors other than concurrent medical conditions determined the survival outcome of elderly OHCA patients (32). These resuscitation-related factors include the patient’s age, initial rhythm, arrest place (public vs. non-public), witnessed status, and bystander CPR (23, 24). Another study by Pun et al. demonstrated that initiating CPR by staff was linked to approximately three-fold odds increase in hospital discharge (2.87, 0.02) and good neurological status at discharge (3.15, 0.03) among patients undergoing HD (33). Given that patients experiencing cardiac arrest have multiple comorbidities regardless of whether they are undergoing HD or not, it seems that resuscitation-related factors such as initial rhythm, witnessed status, and the timing of bystander CPR and AED use may have a more significant impact on survival than comorbidities in this study population.

One important consideration in interpreting the results of this study is the role of various comorbidities and in-hospital interventions following OHCA. While our study did not find a significant impact of undergoing HD on survival outcomes, it is crucial to highlight the complex interplay of factors that influence these outcomes. For instance, the presence of comorbidities such as hypertension and diabetes which are common in populations may not always act as independent risk factors. Instead, the survival outcomes may be more strongly influenced by resuscitation related factors such as initial rhythm or CAG. Regarding the two survivors from the HD group who achieved favorable neurological outcomes, their average age was 65.0 years, and one patient experienced a witnessed arrest. Both individuals received bystander CPR, and their initial rhythm was classified as shockable. Upon arrival at the hospital, they both underwent CAG followed by percutaneous coronary intervention.

### Limitations

This study has several limitations that must be acknowledged. First, our study focused on OHCA patients in the Ulsan region, potentially restricting the applicability of our findings to diverse patient populations across different regions. However, the standardized nature of the EMS system across South Korea, being government-based, may help mitigate potential differences within the country. Second, while we tentatively focused on cardiac arrest patients as our study population, we lacked precise information about the specific cause of the arrest. Due to the limited practice of post-mortem examinations in South Korea, the exact cause of arrest for HD patients remains unknown. Third, the notable difference in sample sizes between the HD and non-HD groups raises concerns about the statistical power and reliability of our findings. We addressed this disparity using both PSM and IPTW. To enhance statistical power, we aimed for optimal homogeneity by including relevant covariates based on the Utstein template for OHCA reporting. For matching, we employed the nearest neighbor algorithm, pairing treated units with control units based on the smallest absolute difference in propensity scores (34). Additionally, we utilized various matching ratios, including both 1:1 and 1:N to maximize our sample size (35, 36). Despite these efforts, the inherent limitations of observational studies remain, and we encourage careful interpretation of our results. It is recommended that future research be conducted to validate our findings through the inclusion of expanded sample sizes. Fourth, we incorporated many variables and used matching methods to improve comparability regarding factors associated with survival. However, we recognize that there is a limitation stemming from unmeasured domains. For example, the total dose of epinephrine may have been associated with AKI in OHCA survivors following resuscitation and patients who underwent ECMO may have initially presented with a poorer prognosis compared to those who did not receive such intervention (35, 36). Moreover, we were unable to provide specific information on the type of HD in the undergoing HD group. Lastly, the 17 EDs varied in their levels of care, which could influence patient outcomes; however, this was not analyzed in the current study.

In conclusion, while undergoing HD might be considered a potential risk factor for cardiac arrest, our study findings did not demonstrate a significant association with the survival outcomes of OHCA patients. Survival did not differ significantly between the HD and non-HD groups. Therefore, it is crucial not to underestimate the importance of resuscitation efforts in HD patients. Instead, in cases where these patients exhibit favorable resuscitation-related factors, healthcare providers should focus on delivering comprehensive and attentive care to optimize treatment outcomes.

## Data Availability

The datasets presented in this article are not readily available because some of datasets used in this study belong to the Ulsan Fire Agency. Requests to access these datasets should be directed to stachy1@paran.com.
